# Validation of Diagnostic Accuracy and Disease Severity Correlation of Chest Computed Tomography Severity Scores in Patients with COVID-19 Pneumonia

**DOI:** 10.3390/diagnostics14020148

**Published:** 2024-01-08

**Authors:** Ivan Brumini, Doris Dodig, Iva Žuža, Klaudija Višković, Armin Mehmedović, Nina Bartolović, Helena Šušak, Đurđica Cekinović Grbeša, Damir Miletić

**Affiliations:** 1 Department of Diagnostic and Interventional Radiology, University Hospital Rijeka, Kresimirova 42, 51000 Rijeka, Croatia; 2 Department of Radiological Technology, Faculty of Health Studies, University of Rijeka, 51000 Rijeka, Croatia; 3 European Telemedicine Clinic S.L., C/Marina 16-18, 08005 Barcelona, Spain; 4 University Hospital for Infectious Diseases “Dr. Fran Mihaljevic”, Mirogojska 8, 10000 Zagreb, Croatia; 5 Department for Infectious Diseases, University Hospital Rijeka, Kresimirova 42, 51000 Rijeka, Croatia

**Keywords:** COVID-19, pneumonia, SARS-CoV-2, thorax

## Abstract

The aim of our study was to establish and compare the diagnostic accuracy and clinical applicability of published chest CT severity scoring systems used for COVID-19 pneumonia assessment and to propose the most efficient CT scoring system with the highest diagnostic performance and the most accurate prediction of disease severity. This retrospective study included 218 patients with PCR-confirmed SARS-CoV-2 infection and chest CT. Two radiologists blindly evaluated CT scans and calculated nine different CT severity scores (CT SSs). The diagnostic validity of CT SSs was tested by ROC analysis. Interobserver agreement was excellent (intraclass correlation coefficient: 0.982–0.995). The predominance of either consolidations or a combination of consolidations and ground-glass opacities (GGOs) was a predictor of more severe disease (both *p* < 0.005), while GGO prevalence alone was not. Correlation between all CT SSs was high, ranging from 0.848 to 0.971. CT SS 30 had the highest diagnostic accuracy (AUC = 0.805) in discriminating mild from severe COVID-19 disease compared to all the other proposed scoring systems (AUC range 0.755–0.788). In conclusion, CT SS 30 achieved the highest diagnostic accuracy in predicting the severity of COVID-19 disease while maintaining simplicity, reproducibility, and applicability in complex clinical settings.

## 1. Introduction

COVID-19 spread rapidly worldwide from China in 2019, putting health care systems globally under great pressure due to patient overflow, insufficient staffing, and scarce medical supplies, evoking grave uncertainties caused by limited understanding of the nature and course of the disease. Assessment of lung involvement on chest CT was one of the first tools proposed for disease severity estimation and outcome prediction, which was pivotal for patient triage and system management in times of the pandemic and limited resources. The European Society of Radiology and European Society of Thoracic Imaging recommended chest CT in COVID-19 patients with respiratory symptoms such as dyspnea and desaturation [1]. Moreover, the STOIC project (Study of Thoracic Computed Tomography In COVID-19) on 10.735 COVID-19 patients showed that the extent of lung involvement evaluated on chest CT was the best predictor of COVID-19 disease severity [2].

At least eight CT scoring systems (CT SS) have been proposed and used for the quantification of lung involvement in acute COVID-19 pneumonia: Total Severity Score [3,4], Overall CT Score [5], Chest CT Score [6,7], Total CT Score [8], Chest CT Severity Score [9], CT Score [10], Radiologic Severity Index [11], and Three-level Chest Severity Score [12]. However, their reproducibility, accuracy to predict disease severity, clinical outcome, and feasibility in a busy clinical setting have not been evaluated and compared. An efficient and applicable scoring system must enable the quantification of lung changes and should be simple and easy to use equally by experienced and inexperienced radiologists and clinicians in a busy clinical setting, with straightforward grading features and definitions, altogether resulting in high reproducibility, excellent interobserver agreement, and high diagnostic accuracy. Analyzing the published CT scoring systems, we expected to find features and patterns that have the greatest value in predicting the clinical course of COVID-19 pneumonia.

The aim of our study was to establish and compare the diagnostic accuracy and clinical applicability of published chest CT severity scoring systems used for COVID-19 pneumonia assessment and to propose the most efficient CT scoring system with the highest diagnostic performance and the most accurate prediction of disease severity.

## 2. Materials and Methods

### 2.1. Participants

After obtaining approval from the institutional review boards, a two-center retrospective study was performed on consecutive hospitalized patients with nasopharyngeal or oropharyngeal RT-PCR [13]-confirmed SARS-CoV-2 and chest CT from March 2020 to December 2021. Patients with previous lung surgery, radiation therapy, or other chronic lung changes were excluded from the analysis. Chest X-ray was the imaging modality of choice and chest CT was not performed in intubated and mechanically ventilated patients; therefore, they were not included in this study. Patient data retrieved from electronic medical records included demographics (age, gender), comorbidities, vaccination status, clinical signs and symptoms, time from illness onset to CT scan, laboratory findings obtained on the day of the CT scan, and duration of hospitalization.

Disease severity was defined as minimal, common, severe, or critical, according to the Diagnosis and Treatment Program of Pneumonia of New Coronavirus Infection (Trial Version 7) [14] recommended by China’s National Health Commission. Patients with minimal disease had mild clinical symptoms and no pulmonary changes on chest CT. Common disease symptoms were fever and respiratory tract infection with features of pneumonia on chest CT. The severe disease group included patients with any of the following criteria: (a) respiratory distress, respiratory rate ≥ 30/min; (b) oxygen saturation ≤ 93% on finger pulse oximeter taken at rest; and (c) arterial partial pressure of oxygen (PaO_2_)/oxygen concentration (FiO_2_) ≤ 300 mmHg (1 mmHg = 0.133 kPa). Critical patients had one of the following conditions: (a) respiratory failure and need for mechanical ventilation; (b) shock; and (c) other organ failure needing intensive care. For the purpose of this study, patients were dichotomized into mild (minimal and common disease) and severe (severe and critical disease) disease severity groups.

### 2.2. CT Scanning

Patients were scanned on a 128-section scanner, Siemens Somatom Definition AS (Siemens Medical Systems, Forcheim, Germany) and a 64-section scanner, FCT Speedia HD (Fujifilm corporation, Tokyo, Japan). All CT examinations were performed in the supine position with maximal possible inspiration, without administration of intravenous contrast material.

### 2.3. Image Evaluation

The images were analyzed, and CT SS calculated independently by two radiologists: a dedicated chest radiologist with 10 years of experience and a general radiologist with 2 years of attending experience. Both radiologists were aware that all patients were COVID-19 positive and were blinded to any other patient details and clinical data. Mediastinal (width 350–450 HU; level 20–40 HU) and lung (width 1200–1600 HU; level −500 to −700 HU) reconstructions were reviewed on a workstation in all three planes. The imaging parameters were set at 1 mm section thickness.

**Figure 1:**
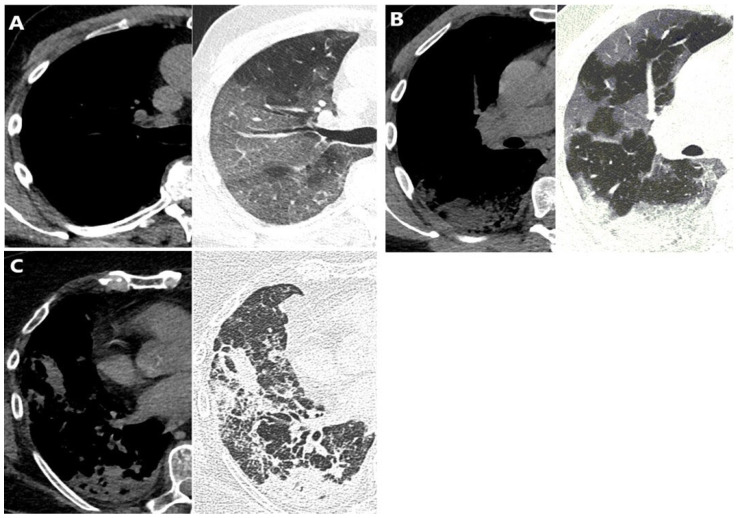
Predominant lung pattern on mediastinal (left) and lung (right) reconstructions of axial CT scans at the level of right lover lobe in three different patients: (**A**) ground-glass opacity, (**B**) mixed pattern (combination of ground-glass opacity and consolidation), (**C**) consolidation.

The presence of the following lung patterns defined by the glossary of terms for chest imaging presented by the Fleischner Society was recorded [15]: ground-glass opacity (GGO), consolidations, air bronchogram, interlobular septal thickening, crazy-paving pattern, pleural thickening, and nodules, as well as presence of lymph node enlargement and pleural and pericardial effusion. Additionally, the readers determined the prevalent lung involvement pattern: GGO, consolidation, or both. Examples of predominant lung patterns are shown in Figure 1.

Furthermore, they quantified the extent of lung involvement by 8 different published CT SSs [3,4,5,6,7,8,9,10,11,12]. The CO-RADS [16], a categorical CT assessment scheme for suspicion of pulmonary involvement of COVID-19, was excluded from our investigation because the sixth category includes patients with RT-PCR-proven SARS-CoV-2, and therefore all our patients would pre-emptively fall into this category.

Based on interim data analysis and feedback from the readers on the applicability and simplicity of the 8 different CT SS applied, we constructed an additional CT scoring system, named CT SS 30, where the readers assessed the percentage of lung involvement by lesions considered to be related to COVID-19 pneumonia on a 5-point scale with the addition of 1 point if consolidation was the predominant lung involvement pattern in each of the lobes.

The number in the abbreviated names of the CT severity scoring systems refers to the maximum score value within each scoring system. The following CT severity score systems were evaluated.

CT SS 20 (“Total severity score“) [3,4].

Points are assigned from 0 to 4 depending on the percentage of parenchymal involvement by GGO, consolidation, or mixed GGO (0, no infiltrate; 1, 1–25%; 2, 26–50%; 3, 51–75%; 4, 76–100%) in each of the 5 lung lobes.

CT SS 24 (“Overall CT score“) [5].

Points are assigned from 0 to 4 depending on the percentage of abnormal parenchymal involvement (0, no infiltrate; 1, 1–24%; 2, 25–49%; 3, 50–74%; 4, 75–100%) in each of the 6 lung zones: upper (lung parenchyma above the carina), middle (lung parenchyma between the carina and the inferior pulmonary vein), and lower (lung parenchyma below the inferior pulmonary vein) lung zone on each side.

CT SS 25 (“Chest CT score“) [6,7].

Points are assigned from 0 to 5 depending on the percentage of involvement of the lung parenchyma by either GGO, crazy paving, or consolidation (0, no infiltrate; 1, <5%; 2, 5–25%; 3, 26–49%; 4, 50–75%; 5, >75%) in each of the 5 lung lobes.

CT SS 35 (“Total CT score“) [8].

The base CT score is assigned on a 5-point scale (as in CT SS 25) based on the extent of lung involvement by GGO with the addition of 1 added point for the presence of a crazy-paving pattern and 2 points for the presence of consolidation (irrespective of the presence of a crazy-paving pattern).

CT SS 40 (“Chest CT severity score“) [9].

Eighteen lung segments are divided into twenty regions, in which the posterior apical segment of the left upper lobe is subdivided into apical and posterior segmental regions, whereas the anteromedial basal segment of the left lower lobe is subdivided into anterior and medial segmental regions. Points are assigned from 0 to 2 depending on the percentage of lung involvement by lung opacities in each region (0, no infiltrate; 1, <50%; 2, ≥50% of lung involvement).

CT SS 48 (“CT score“) [10].

Each lung is divided into 3 zones (as in CT SS 24), with an additional division of each zone into an anterior and posterior segment in relation to the vertical line through the midpoint of the diaphragm in the coronal plane. The points for the extent of lung involvement by GGO, consolidation, both GGO and consolidation, or crazy paving are assigned from 0 to 4 (as in CT SS 24) for each of the 12 lung zones.

CT SS 72 (“Radiologic Severity Index”) [11].

Each lung is divided into 3 zones (as in CT SS 24) and points are assigned from 1 to 3 depending on the predominant pattern: (1) normal attenuation of the parenchyma; (2) GGO; (3) consolidation. That value is multiplied by a factor representing the extent of parenchymal involvement by GGO, consolidation, or nodular opacities scored from 0 to 4 (as in CT SS 20).

CT SS 96 (“3-level chest severity score“) [12].

Similarly to CT SS 72, each lung is divided into 3 zones, and the extent of lung involvement by GGO, crazy paving, and consolidation in each zone is estimated on a scale from 0 to 4. Furthermore, the predominant pattern is scored from 1 to 4: (1) normal lung parenchyma; (2) at least 75% GGO/crazy-paving pattern; (3) combination of GGO/crazy-paving pattern and consolidation if each has less than 75% involvement; and (4) at least 75% consolidation. The two scores (the extent and pattern of pulmonary involvement) in each lung zones are multiplied by each other and all the scores in each of the 6 lung zones are added together.

CT SS 30.

Points are assigned from 0 to 5 for each lung lobe depending on the percentage of lung involvement by GGO, consolidation, or other opacities considered to be related to viral pneumonia (0, no infiltrate; 1, <5%; 2, 5–25%; 3, 26–49%; 4, 50–75%; 5, >75%) with 1 point added if consolidation is the predominant lung pattern. Maximum score for each lung lobe is 6.

All the scoring systems are CT-based. However, they all approach the assessment and quantification of lung involvement in COVID-19 pneumonia differently, in an attempt to correlate the scores with disease severity by applying various scales of the extent of lung involvement, dividing the lungs into lobes, zones, or regions, and differently adding additional points for the presence of patterns more specific to COVID-19 pneumonia, such as GGO, crazy paving, or consolidation.

### 2.4. Statistical Analysis

Statistical analysis was performed using Statistica for Windows, version 14.0.0.15 (Statsoft, Inc., Tulsa, OK, USA) and MedCalc 20.116 (MedCalc Inc., Mariakerke, Belgium). The normality of distribution of quantitative variables (age, length of hospital stay, laboratory test results, and CT scores) was evaluated by the Kolmogorov–Smirnov test with Lilliefors correction. Although some of these variables were not normally distributed, we used mean ± standard deviation (SD) to present all data because it allows for easier understanding and comparison with other studies. Two patient groups were compared by the Mann–Whitney test or *t*-test for independent samples, respectively. To test the differences between the groups according to gender, symptoms, or comorbidities, we used the Pearson χ^2^-test. The analysis of the presence and the type of lung patterns was performed using the Pearson χ^2^-test or Fisher exact test. Inter-rater reliability was established by the intraclass correlation coefficients (ICC) grading system developed by Koo TK and Li MY (poor: <0.40; fair: 0.40–0.59; good: 0.60–0.74; excellent correlation: 0.75–1.00) [17]. The correct ICC form for inter-rater reliability included a two-way random-effects model and absolute agreement.

Association between variables was calculated by Spearman’s rank or Pearson’s correlation coefficient. The impact of the predominant lung pattern on the odds ratio of disease severity was assessed by logistic regression. Receiver operating characteristic (ROC) analysis was used to calculate the ROC plot and overall sensitivity and specificity of each CT scoring system. The area under the ROC curve (AUC) was a measure of how well the CT scoring system discriminates between mild and severe disease groups. All statistical values were considered significant at the level of *p* < 0.05.

## 3. Results

### 3.1. Patient Characteristics

Two hundred and eighteen patients were enrolled, with male predominance (male: female; 138:80) and mean age ± SD of 62 ± 15 years. A total of 105 patients (48%) were within the mild and 113 (52%) within the severe disease group. The demographic characteristics, symptoms, and comorbidities of the patients are shown in Table 1.

Patients with severe disease were significantly older than patients with mild disease (68 vs. 55 years, *p* < 0.001), with a similar gender-based distribution (*p* = 0.208). Severely ill patients stayed in the hospital significantly longer than patients with mild disease, with an average hospitalization duration of 17 days (range 7–27 days), compared to 13 days (range 7–19 days), respectively (*p* = 0.001). The most common symptoms in all patients were fever (85.8%), cough (81.2%), and myalgia/fatigue (68.3%). Significantly more patients with hypertension and COPD had more a severe course of illness (*p* = 0.001, and *p* = 0.014, respectively), while other comorbidities were similarly distributed among groups. There were only 20 (20/218, 9.2%) vaccinated patients in our sample, with the different types of vaccine as follows: Pfizer (13 patients), Moderna (2 patients), Janssen (2 patients), AstraZeneca (1 patient), and unknown vaccine (2 patients). Furthermore, vaccinated and unvaccinated patients were evenly distributed among groups with mild and severe disease (13 vs. 7, respectively; *p* = 0.114). The mortality rate was 6.8% (15/218 patients). A comparison of clinical signs and laboratory findings between groups with mild and severe COVID-19 disease is shown in Table 2.

### 3.2. Pattern Distribution

The distribution of the most common CT lung patterns is shown in Table 3. Consolidations were significantly more frequent in severe cases (*p* = 0.023), while the incidence of GGOs and mixed patterns did not differ between the groups (Table 3). Additionally, logistic regression showed that the predominance of either consolidations or the mixed pattern was a predictor of disease severity (both *p* < 0.005), while GGO alone was not (*p* = 0.128), as detailed in Table 4.

### 3.3. Reproducibility and Diagnostic Accuracy

Interobserver agreement was excellent for all CT severity scores, ranging from 0.982 to 0.995. There was a high correlation between all CT severity scores, ranging from 0.848 to 0.971 (all *p* < 0.001). The correlation between CT severity scores and disease severity was lower, ranging from 0.570 to 0.601, although it showed statistical significance (all *p* < 0.001). ROC analysis demonstrated that the diagnostic performance of all CT severity scores was comparable, with good predictive values and an AUC between 0.75 and 0.81. Detailed results are displayed in Table 5.

**Figure 2:**
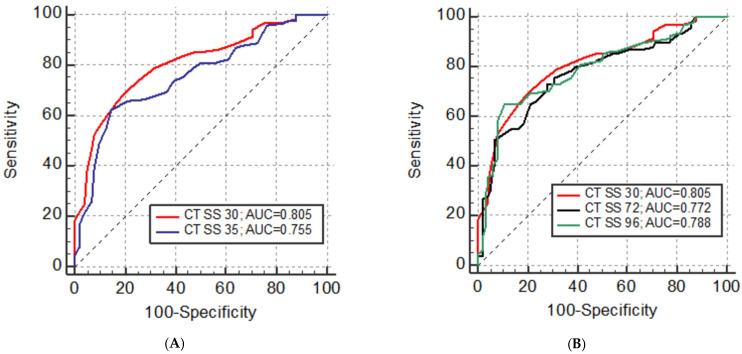
(**A**) Diagnostic accuracy and discriminative capacity of CT severity scores (CT SSs) to differentiate mild from severe COVID-19 disease estimated by receiver operating characteristic (ROC) analysis for CT SS 30 and CT SS 35. (**B**) Diagnostic accuracy and discriminative capacity of CT severity scores (CT SSs) to differentiate mild from severe COVID-19 disease estimated by receiver operating characteristic (ROC) analysis: CT SS 30, CT SS 72, and CT SS 96.

Overall, CT SS 30 (AUC = 0.805) demonstrated the highest diagnostic accuracy compared to all the other CT SS systems. CT SS 30 was significantly superior to the other five CT SS systems in discriminating patients with mild from those with severe disease [CT SS 20 (AUC = 0.768, *p* < 0.001), CT SS 24 (AUC = 0.764, *p* = 0.005), CT SS 25 (AUC = 0.756, *p* < 0.001), CT SS 35 (AUC = 0.755, *p* < 0.001), and CT SS 48 (AUC = 0.771, *p* = 0.022)], without significant difference compared to CT SS 40, CT SS 72, and CT SS 96. The respective ROC curves are presented in Figure 2. At the cut-off of 13 points, the sensitivity of CT SS 30 for correctly establishing disease severity was 69%, with 80% specificity.

## 4. Discussion

GGO is the most common CT feature of COVID-19 pneumonia [18,19,20]. Consistently with previous studies, GGO and consolidations were the most prevalent lung patterns in our entire cohort (86% and 69%, respectively) as well as in patients with both mild and severe disease, followed by interlobular septal thickening, air bronchogram, and crazy-paving patterns [20,21]. However, consolidation was the only pattern significantly more prevalent in patients with severe COVID-19 pneumonia. Moreover, the probability of developing severe COVID-19 disease was twice as high in patients with lung consolidations compared to those with only GGO or mixed changes, which is consistent with prior studies [18,22]. As consolidation represents the most advanced form of acute pneumonic lung infiltration, it was anticipated to be more frequent in severely ill patients [23]. However, in our study, it was GGO that was in fact the most common finding, seen in 45% of patients with severe COVID-19 pneumonia, followed by consolidations in 39.5% of severe cases. This indicates that consolidations might be a sole imaging discriminating factor for predicting acute disease severity, patient outcome, and patient management. It is furthermore expected that the extent of lung abnormalities influences patient condition and the course of disease and, therefore, many different approaches to quantify the severity of lung involvement were proposed during the start of COVID-19 pandemic [3,4,5,6,7,8,9,10,11,12]. Based on our lung pattern data analysis and evaluation of previously published CT SS systems, we constructed a CT severity score with the aim to emphasize consolidations as a presumed morphological disease severity discriminator while keeping the scoring method simple, fast, intuitive, easy to remember, and applicable to radiologists and clinicians of different backgrounds. The extent of lung involvement was quantified on a 5-point scale and 1 point was added if consolidation was the predominant pattern in each lung lobe. A 5-point scale with a 5% threshold allows for better discrimination of likely asymptomatic patients with only minimal lung changes from those with up to one-quarter of lobe infiltration (1 point for <5% and 2 points for 5–25% involvement on a 5-point scale vs. 1 point for 1–25% lobe involvement on a 4-point scale, respectively), as demonstrated by Inoue et al. [24]. An anatomical lung division into lobes rather than zones is clearer and more intuitive to radiologists and clinicians as it is routinely used in reporting of any other lung pathology on chest CT. Only three other published CT severity scores, CT SS 35, CT SS 72, and CT SS 96, factored in the pattern of parenchymal infiltration, while the others only evaluated the extent of lung involvement [8,11,12]. The CT SS 96 scoring system seemed complicated and time-consuming, as it requires the reader to simultaneously keep in mind the borders of six non-anatomical lung zones while estimating the percentage correlation of three different patterns (GGO, crazy paving, and consolidation) and finally having to multiply and add the scores from each lung zone [12,25]. CT SS 72 is simpler, as points are assigned only for the presence of GGO or consolidation; however, the lungs are also divided into six non-anatomical zones [11]. CT SS 35 is the most similar to our proposed CT SS 30 with the only difference being that it takes into account both crazy paving, with 1, and consolidation, with 2 extra points in each of the lobes [8].

CT SS 30 had the highest diagnostic accuracy (80.5%) in discriminating mild from severe COVID-19 disease compared to all the other proposed scoring systems (range 75.5–78.8%). At the same time, it is the simplest and fastest to use with excellent inter-rater agreement (ICC = 0.991). Interestingly, CT SS 35, which is the most similar scoring system (the only difference is that it additionally scores both crazy paving and consolidation), showed the lowest overall diagnostic accuracy of 75.5%, while the more complex CT SS 96 and CT SS 72 were the second and third highest performing scoring systems (79% and 77%, respectively). It can be assumed that factoring crazy paving into CT SS 35 confounded its accuracy in determining the severity of acute COVID-19 pneumonia, as crazy paving does not necessarily represent the progression of GGO into consolidation but may also be a sign of healing or of the development of chronic changes [26,27]. Moreover, both other highest scoring systems, CT SS 72 and CT SS 96, did not additionally score the crazy paving pattern (CT SS 96 only scored the combination of GGO and crazy paving together). However, these scoring systems assigned extra scores for both GGO and consolidation, and it could similarly be assumed that additional points for the presence of GGO decrease the diagnostic accuracy of the scoring system to discriminate mild from severe disease, as GGO is the most prevalent and not the most specific lung infiltration pattern in severe COVID-19 pneumonia. Additionally, discrepant results of CT SS 30 versus CT SS 72 and CT SS 96 may be due to different lung segmentation (five lobes versus six lung zones, respectively) and different ways of calculating the scores and pondering the predominant lung involvement patterns. Overall, CT SS 72 and CT SS 96 were both deemed substantially more complex to use and less accurate compared to our proposed CT SS 30. Our findings and observations show that a clinically relevant scoring system can be both simple and efficient if the key discriminating feature (consolidation in the case of acute COVID-19 pneumonia) is recognized and confirmed based on adequate research and data analysis.

The overall diagnostic accuracy and AUC in our study for all CT SSs were between 0.75 and 0.81 and was lower compared to previously reported data, where they range from 0.81 to 0.92 [4,9,11,12,24,25]. However, we find our results to be more reliable due to the substantially larger sample size of 218 patients (compared to the largest cohort of 165 patients [12]) and the even distribution of patients with mild and severe COVID-19 disease, while severely ill patients constituted a minority in previous studies, from 10% to 39% of the entire cohort [4,7,9,12,24,25]. All the analyzed CT scoring systems showed excellent interobserver agreement without any significant difference between simple and more complex methods, which is in keeping with prior studies [24,25]. However, it is desirable for any method to be as simple as possible without compromising its diagnostic accuracy and CT SS 30 proved to have all these qualities, compared to any other severity score.

Two hundred and eighteen patients in our cohort were equally distributed among groups with mild and severe COVID-19 disease. Observational studies report that older age, male sex, and comorbidities are factors associated with higher disease severity and mortality, with men having more than twice as high a mortality rate compared to women [28]. The patient cohort in our study is representative of the whole population, as more men than women had a severe form of disease (55% vs. 46%, respectively) and patients with severe disease were significantly older than those with mild symptoms (mean age 68 vs. 55, respectively) [29,30]. However, only hypertension, COPD, and dyspnea proved to be more prevalent in patients with severe disease [18,28]. Diarrhea, dysgeusia, nausea/vomiting, and hyposmia/anosmia were more prevalent in patients with mild COVID-19 disease. CRP, body temperature, LDH, leukocytes, and neutrophils were significantly higher, while lymphocytes and oxygen saturation were significantly lower in severe cases. These results are in accordance with previously reported findings [29,31,32].

The retrospective design of our study relatively limits its power to identify prognostic factors for disease severity and patient outcome. Furthermore, participant sampling depended on the clinical practice adopted at the time in two major national centers where this study was conducted. Therefore, the results might not be applicable or relevant to other centers or countries as clinical practice and availability of CT might be very different. Lastly, there was no long-term follow-up after patient discharge to evaluate the relation of CT severity scores and long-term COVID-19 sequelae.

The authors recognize that CT might no longer be the most relevant factor in COVID-19 management. However, this might exactly be due to lack of validated, robust, and clinically relevant quantifying methods for determining disease severity and predicting patient outcomes. This study shows that a simple, fast, non-invasive semi-quantitative imaging method can be established and can serve as a significant tool in patient management even in a busy workflow, difficult conditions, and lack of specialized staff.

In conclusion, the CT SS 30 system, as designed based on performance analysis of other scoring methods, achieved the highest diagnostic accuracy in predicting the severity of COVID-19 disease while maintaining simplicity and reproducibility, all crucial attributes for its relevance, applicability, and acceptance in the complex clinical setting.

## Figures and Tables

**Table 1:** Demographic characteristics, symptoms, and comorbidities of COVID-19 patients.

	All (*N* = 218)	Mild Disease (*N* = 105)	Severe Disease (*N* = 113)	*p* Value
	Mean ± SD			
Age (years)	62 ± 15	55 ± 15	68 ± 12	<0.001 *
Length of stay in hospital (days)	15 ± 9	13 ± 6	17 ± 10	0.001 *
	*N* (%)			
Gender				
Male	138 (63.3)	62 (28.4)	76 (34.9)	0.208
Female	80 (36.7)	43 (19.7)	37 (16.9)	
Symptoms				
Fever	187 (85.8)	92 (42.2)	95 (43.6)	0.453
Cough	177 (81.2)	83 (38.0)	94 (43.2)	0.434
Myalgia/fatigue	149 (68.3)	74 (33.9)	75 (34.4)	0.515
Headache	70 (32.1)	39 (17.9)	31 (14.2)	0.125
Dyspnea	92 (42.2)	31 (14.2)	61 (27.9)	<0.001 *
Diarrhea	57 (26.1)	37 (16.9)	20 (9.2)	0.003 *
Chest pain	20 (22.5)	12 (13.5)	8 (9.0)	0.871
Nausea/vomiting	41 (18.8)	28 (12.8)	13 (5.9)	0.004 *
Hyposmia/anosmia	41 (18.8)	30 (13.8)	11 (5.1)	<0.001 *
Dysgeusia	44 (20.2)	29 (13.3)	15 (6.9)	0.008 *
Comorbidities				
Hypertension	122 (55.9)	47 (21.6)	75 (34.4)	0.001 *
Diabetes	40 (18.3)	16 (7.3)	24 (11.0)	0.252
Malignancy	27 (12.4)	13 (5.9)	14 (6.4)	0.998
Asthma	16 (7.3)	11 (5.0)	5 (2.3)	0.086
COPD	16 (7.3)	3 (1.4)	13 (5.9)	0.014 *
Smoking	10 (4.6)	5 (2.3)	5 (2.3)	0.905
Osteoporosis	6 (2.7)	2 (0.9)	4 (1.8)	0.460

* Indicates statistically significant results. SD, standard deviation; COPD, chronic obstructive pulmonary disease.

**Table 2:** Clinical signs and laboratory findings of COVID-19 patients.

Laboratory Findings	All (*N* = 218)	Mild Disease (*N* = 105)	Severe Disease (*N* = 113)	*p* Value
	Mean ± SD			
Temperature (°C)	37.5 ± 0.9	37.4 ± 0.8	37.7 ± 0.9	0.039 *
SpO_2_ (%)	92.8 ± 5.5	95.9 ± 1.8	90.9 ± 6.3	<0.001 *
PaO_2_ (kPa)	10.6 ± 2.5	11.7 ± 2.3	8.4 ± 0.9	<0.001 *
CRP (mg/L)	82.5 ± 72.2	55.0 ± 52.3	110.2 ± 87.5	<0.001 *
D-dimer (mg/L)	2.3 ± 5.4	2.0 ± 2.8	3.5 ± 7.3	0.099
LDH (U/L)	286.8 ± 128.0	251.4 ± 98.0	318.0 ± 124.8	<0.001 *
Erythrocytes (10^12^/L)	4.57 ± 0.63	4.65 ± 0.58	4.46 ± 0.69	0.165
Thrombocytes (10^9^/L)	228.0 ± 96.14	216.40 ± 77.65	238.74 ± 108.84	0.090
Leukocytes (10^9^/L)	7.44 ± 3.99	6.51 ± 2.65	8.29 ± 4.76	0.001 *
Neutrophils (10^9^/L)	5.54 ± 3.87	4.31 ± 2.32	6.66 ± 4.60	<0.001 *
Lymphocytes (10^9^/L)	1.15 ± 0.63	1.45 ±0.68	0.88 ± 0.44	<0.001 *
Eosinophils (10^9^/L)	0.06 ± 0.29	0.10 ± 0.42	0.02 ± 0.06	0.079
Procalcitonin (µg/L)	0.43 ± 1.57	0.28 ± 0.47	0.49 ± 1.88	0.504

* Indicates statistically significant results. SD, standard deviation; SpO_2_, arterial oxygen saturation; PaO_2_, arterial partial pressure of oxygen; CRP, C-reactive protein; LDH, lactate dehydrogenase.

**Table 3:** Distribution of most common lung patterns and secondary CT findings in COVID-19 patients.

Lung Pattern	All	Mild Disease	Severe Disease	*p* Value
	*N* (%)			
GGO	187 (85.8)	89 (40.8)	98 (44.9)	0.687
Mixed ^†^	102 (46.8)	52 (23.8)	50 (22.9)	0.435
Consolidation	151 (69.2)	65 (29.8)	86 (39.5)	0.023 *
Air bronchogram	112 (52.8)	47 (22.1)	65 (30.7)	0.108
Interlobular septal thickening	138 (63.3)	60 (27.5)	78 (35.8)	0.062
Crazy-paving pattern	114 (52.3)	48 (22.0)	66 (30.3)	0.060
Pleural thickening	52 (23.8)	27 (12.4)	25 (11.4)	0.534
Nodules	28 (12.8)	10 (4.6)	18 (8.2)	0.158
Lymph node enlargement	60 (27.5)	26 (11.9)	34 (15.6)	0.378
Pleural effusion	45 (20.6)	16 (7.3)	29 (13.3)	0.052
Pericardial effusion	6 (2.8)	0 (0)	6 (2.8)	0.017 *

* Indicates statistically significant results. GGO, ground-glass opacity. ^†^ Mixed = combination of ground-glass opacity and consolidation.

**Table 4:** Impact of predominant lung pattern on COVID-19 disease severity.

Predominant Lung Pattern	Odds Ratio	95% CI
GGO	1.21	0.87–1.48
Mixed ^†^	1.40	1.06–1.86
Consolidation	2.54	1.78–3.61

CI, confidence interval; GGO, ground-glass opacity. ^†^ Mixed = combination of ground-glass opacity and consolidation.

**Table 5:** ROC analysis and diagnostic accuracy of CT severity scores (CT SS).

CT SS	AUC	SE	95% CI	*p*	Cut-Off	Sensitivity %	Specifity %
CT SS 20	0.768	0.032	0.706 to 0.822	<0.001 *	>9	61.9	84.6
CT SS 24	0.764	0.032	0.702 to 0.819	<0.001 *	>10	68.1	79.0
CT SS 25	0.756	0.032	0.694 to 0.812	<0.001 *	>13	62.8	80.2
CT SS 30	0.805	0.029	0.747 to 0.856	<0.001 *	>13	69.0	80.0
CT SS 35	0.755	0.032	0.692 to 0.811	<0.001 *	>20	61.9	85.7
CT SS 40	0.781	0.031	0.721 to 0.834	<0.001 *	>19	74.3	72.4
CT SS 48	0.771	0.032	0.710 to 0.825	<0.001 *	>18	73.5	78.1
CT SS 72	0.772	0.032	0.710 to 0.826	<0.001 *	>20	72.6	72.4
CT SS 96	0.788	0.031	0.727 to 0.840	<0.001 *	>28	64.6	82.7

* Indicates statistically significant results. AUC, area under the curve; SE, standard error; CI, confidence interval; CT SS, CT severity score.

## Data Availability

All data generated or analyzed during this study are included in this article. Further enquiries can be directed to the corresponding author.
